# Jellyfish Bioactive Compounds: Methods for Wet-Lab Work

**DOI:** 10.3390/md14040075

**Published:** 2016-04-12

**Authors:** Bárbara Frazão, Agostinho Antunes

**Affiliations:** 1 CIIMAR/CIMAR, Interdisciplinary Centre of Marine and Environmental Research, University of Porto, Rua dos Bragas 177, 4050-123 Porto, Portugal; bfrazao@ciimar.up.pt; 2 Department of Biology, Faculty of Sciences, University of Porto, Rua do Campo Alegre, 4169-007 Porto, Portugal

**Keywords:** jellyfish, toxin, methods, proteomic, transcriptomic, deep sequencing

## Abstract

The study of bioactive compounds from marine animals has provided, over time, an endless source of interesting molecules. Jellyfish are commonly targets of study due to their toxic proteins. However, there is a gap in reviewing successful wet-lab methods employed in these animals, which compromises the fast progress in the detection of related biomolecules. Here, we provide a compilation of the most effective wet-lab methodologies for jellyfish venom extraction prior to proteomic analysis—separation, identification and toxicity assays. This includes SDS-PAGE, 2DE, gel chromatography, HPLC, DEAE, LC-MS, MALDI, Western blot, hemolytic assay, antimicrobial assay and protease activity assay. For a more comprehensive approach, jellyfish toxicity studies should further consider transcriptome sequencing. We reviewed such methodologies and other genomic techniques used prior to the deep sequencing of transcripts, including RNA extraction, construction of cDNA libraries and RACE. Overall, we provide an overview of the most promising methods and their successful implementation for optimizing time and effort when studying jellyfish.

## 1. Introduction

Many investigations in marine animals target the study of bioactive compounds for various purposes including the discovery of novel drugs, nutritional supplements and applications for industrial biotechnology [1]. This can be accomplished *in vivo* and *in vitro*, for example by the direct study of produced venoms or, more indirectly, by deep sequencing and *in silico* analyses of the genome of these animals. Marine bioprospecting in tropical and sub-tropical species has resulted in the discovery of several bioactive compounds which are of great benefit to human medicine, including cancer therapy, vascular diseases and infectious viral diseases such as AIDS. For example, some soft corals have been exploited for the isolation of products with anti-inflammatory activities [2]. Bioprospecting in species found in Arctic and sub-Arctic waters has allowed us to discover products adapted to extreme environmental conditions such as low temperatures or seasonal lighting [3,4,5].

Jellyfish (or medusae) comprise a group of free-swimming animals that belong to the phylum Cnidaria and have representatives in the Schyphozoa, Cubozoa and Hydrozoa classes. Cnidarians have special cells, cnidocytes, which gave the name to the phylum and contain a special organelle called the cnidocyst. Research on cnidocysts can have several purposes, but often they are studied for their toxic potential. Nonetheless, toxicity is not exclusively associated to this kind of cell [4]. In fact neurotoxins are also associated with ectodermal gland cells [6]. Therefore, exhaustive studies often consider not only the nematocyst venom but also the toxicity of the animal’s body parts [6].

The direct study of the jellyfish venom requires several common steps. Firstly, the protein sample should be obtained by isolating the nematocysts and releasing its venom from the interior of the organelle (or from the tissue samples). Then, the purification of the crude extract may rely on several methods including gel electrophoresis, gel chromatography, and HPLC, among others. For better quality results, liquid chromatography LC-MS or HPLC may be used. Western blot is a technique allowing the detection of specific proteins in a sample which is used when the toxin is more or less well characterized. Hemolytic, antimicrobial and protease activity assays, among others, are also common approaches for these studies. When the interest is on amino acid sequence identification there are direct methods, such mass spectrometry, or indirect methods, such as RNA extraction, construction of cDNA libraries, Northern blot analysis and RACE amplifications, which will lead to the nucleotide sequence of the gene.

Besides this direct study of the venom, the protein content of a species can also be inferred by sequencing its transcriptome or genome. The transcriptome study can be simple since it allows us to more directly characterize protein-coding genes. This review also systematizes the deep sequencing platforms used in cnidarians for optimizing their appropriate use and choice.

Given the broadness of this review, some contents, while relevant, will not be discussed in detail (e.g., toxicity assays in animals, data handling and bioinformatics analyses—*in silico* lab work). Overall, this review will enable the reader to choose the most appropriate methods for the evaluation of bioactive compounds in jellyfish by using either genomic, proteomic or deep sequencing platform tools.

## 2. Proteomics in Jellyfish

Here we describe how to prepare the biological material for proteomic analysis, including the appropriate kits, type of columns and technologies needed for obtaining and processing crude extracts. Bioassays requiring highly specialized facilities or authorizations will not be contemplated. Sea anemones and jellyfish procedures were reviewed previously in 2012 [6] and 2004 [7], respectively.

Here we reviewed relevant information on several jellyfish species including *Aurelia aurita* [8,9,10,11], *Carybdea alata* [12], *Carybdea rastoni* [13], *Chironex fleckeri* [14,15,16,17,18,19], *Chiropsalmus quadrigatus* [20], *Chiropsalmus* sp. [14], *Chiropsella bronzie* [17], *Cyanea capillata* [21,22], *Cyanea nozakii* [11,23,24], *Nemopilema nomurai* [11,25,26], *Pelagia noctiluca* [27,28,29,30,31,32,33], *Rhopilema esculentum* [11,34,35], *Rhopilema nomadica* [36], *Stomolophus meleagris* [37,38,39,40,41] and deep-sea jellyfish [42]. Apart from the true jellyfish, there are also Hydrozoa members that have different evolutionary histories [43] and are colonial free-swimming organisms resembling jellyfish. *Olindias sambaquiensis* [44,45] and *Physalia physalis* [46] are two of such examples. When considering jellyfish sample collection, the most important step is to quickly preserve proteins from any kind of degradation. For that purpose, all the procedures should be kept in ice to avoid loss of efficacy [14], or they should proceed as explained by the authors. Jellyfish can be transported live to the laboratory in plastic buckets with seawater [44] or processed in the field. Tentacles can be immediately processed and stored in seawater at 4 °C [17] prior to nematocyst extraction. Alternatively, the tissues can be immediately stored at −20 °C [23]. The transport of jellyfish to the laboratory facility should consider the use of ice [11] or dry ice [12,13,20]. Dry ice [21] or lyophilized material [47] is also suitable for shipping material. Another option is to snap-freeze tentacles in liquid nitrogen and store them at −80 °C [12,13,37,38] until use.

### 2.1. Nematocyst Extraction

#### 2.1.1. Autolysis

The nematocyst extraction can be accomplished using several protocols. One of the most commonly used methods is that proposed by Bloom *et al.* [48], which requires refrigerating the tentacles in two volumes of seawater for one to four days. Once a day the containers should be vigorously shaken and an aliquot filtered through a fine kitchen sieve. The decision on whether or not to continue digestion of individual samples is based on microscopic examination of the tentacles and the filtered sediment. If digestion is to be continued, the original fluid is decanted, replaced with fresh seawater, and the filtered material washed with seawater, allowing three hour-long settling periods under refrigeration. The final sediments are then lyophilized and stored at −80 °C until use.

Several works [17,19,26,47] used this well-established method (or with slight adaptations). Feng *et al.* [23] has frozen the tentacles placed in 5× vol. of fresh seawater at 4 °C and set the autolysis for four days. The resulting suspension was filtered through a fine sieve (54 mm). The sediments (where most of the nematocysts are contained) were then collected and washed several times with 0.9% NaCl solution. Xiao *et al.* [21] set the mass:volume ratio to 1:1. The mixture was stirred for 30 min twice daily and the autolyzed mixture was centrifuged thrice. Nagai *et al.* [12] isolated the nematocysts using a modification of the Burnett method [49]. After four days, the sample was filtered through a nylon stocking and the nematocysts in the filtrate were allowed to settle in a 50 mL conical centrifuge tube. More recently, the method has been simplified and performed faster [24,37] with an overnight autolysis at 4 °C, and afterwards the tentacles are stirred for 10 min and filtered through a plankton net (approximately 280 µm) to remove most of the tentacle debris. Brinkman *et al.* [15] purified the nematocysts from tentacle debris by centrifugation in a discontinuous Percoll gradient diluted with 35 g/L NaCl.

In other works [27,28,29,30,32], the nematocysts were isolated as described by Salleo *et al.* [50]. Briefly, the oral arms were excised and submerged in distilled water for 2 h at 4 °C. After a complete detachment of the epidermis, the tissue was removed from the suspension, containing both epidermis and undischarged nematocysts (organoids) deriving from the osmotic rupture/lysis of nematocytes. The nematocysts that were still attached to the epidermal tissue were separated by stirring. The nematocyst suspension was repeatedly washed in distilled water and filtered through plankton nets to remove most of the tissue debris, and then centrifuged. In a similar way, Lee *et al.* [11] and Kang *et al.* [25,51] gently swirled the tentacles with distilled water, and then stood still for 1–2 h to remove debris and seawater. After decanting the supernatant, tentacle pellets settled down at the bottom were resuspended in about a 2× vol. of distilled water and shaken vigorously for 3 min. However, in this procedure the detached nematocysts were separated by filtering the tentacle preparation through four layers of medical gauze.

In the study of the tentacle venom fraction from the jellyfish *Aurelia aurita* [9], cold deionized water and a protease inhibitor cocktail was used for nematocyst extraction. Previously, Gusmani *et al.* [36] used the scapular and mouth filaments suspended in cold sterilized distilled water containing 1 M glycerol for 2 h at 5 °C, and then stirred it. The resulting suspension was filtered through a plankton net and the mesogleal component discarded. After centrifugation the supernatant was discarded and the pellet was resuspended in the same medium. The procedure was repeated twice and the final suspension containing undischarged nematocysts was deep-frozen for further use. In brief, autolysis can be performed in fresh seawater, artificial seawater, filtered seawater or reverse-osmosis purified water to different concentrations of saline solution [10,14,22].

#### 2.1.2. Alternative to Autolysis

As an alternative to autolysis with different types of water, Weston *et al.* [44] isolated the nematocysts using a modification of the Weber *et al.* [52] method. The tentacles were gently homogenized in a pestle and mortar in cold SuFi (sucrose + Ficoll-Paque Plus) solution. The material was kept at 4 °C for 30 min and then passed through a 2 mm diameter sieve. The sample was centrifuged and the supernatant containing debris and cell fragments was removed. The pellet containing intact nematocysts was carefully suspended and washed three times in cold SuFi solution. The final material was submitted, after microscopic inspection, for lyophilization. In a different approach, Ovchinnikova *et al.* [8] excised pieces of *Aurelia aurita* and placed them for extraction into 5% of acetic acid. The obtained extract was consecutively passed under pressure, three times, through a stirred ultrafiltration cell and dried in vacuum. On the other hand, Radwan *et al.* [47] attempted to dissociate the nematocysts from both tentacle tissue and dinofagellates by renograffin density gradient centrifugation according to Calon *et al.* [53].

The simpler the method, the better, as long as it performs well. Indeed, simpler methods are faster and less susceptible to errors. Autolysis overnight at 4 °C, tentacles stirred for 10 min and filtered through a plankton net are suitable to obtain a nematocyst solution.

### 2.2. Venom Extraction

#### 2.2.1. Sonication

For venom extraction several protocols can be used. Venom can be obtained by sonication of the nematocyst suspension in cold extraction buffer [24,27,28,29,30,37,38,39] followed by centrifugation. Bloom *et al.* [48] proposed a widely used protocol. Freeze-dried samples are resuspended in 1:6 with cold deionized water and sonicated for three 20 s periods. Sonications are intercalated with cooling periods of at least 1 min on ice. The degree of nematocyst rupture is examined microscopically. The suspension is clarified by centrifugation at 20,000× *g* for 1 h at 4 °C, being the supernatant composed of the nematocyst proteins.

Nematocyst sonication can be performed in the presence of different buffers. Weston *et al.* [44], disrupted in a sonic bath, freeze-dried nematocysts in TEAB (Triethylammonium bicarbonate buffer) whereas Gusmani *et al.* [36] sonicated the nematocysts in acetate buffer containing NaCl and the protease inhibitors benzamidine and iodoacetic acid. Nagai *et al.* [12,13] sonicated nematocysts and frozen tentacles in a phosphate buffer solution.

#### 2.2.2. Nematocyst Mechanical Disruption

##### Glass Beads

Extraction of venom according to Carrette and Seymour [7] using glass beads in an ice-cold (4 °C) solution has been adopted by several authors [11,14,25,26]. The samples incubated with PBS solution were shaken in a mini-bead mill with intervals five times with intermittent cooling on ice. The venom extract was then transferred to a new Eppendorf^®^ tube and centrifuged. Both the supernatant and the venom were used. Winter *et al.* [17,18] applied this method, but replaced the PBS solution with distilled water. In a different approach, Brinkman *et al.* [7,15] resuspended the nematocysts in ice-cold buffer (MOPS, NaCl and protease inhibitors) and ruptured them using 0.5 mm glass beads. Nematocyst disruption was monitored microscopically and >90% rupture of nematocysts was achieved with four to six cycles of homogenization. Crude nematocyst extracts were clarified by centrifugation. In the case of Feng *et al.* [23] lyophilized nematocysts were placed into screw-top vials with Tris-HCl buffer and glass beads. Samples were shaken four times in a mini–bead beater with intervals on ice. The venom samples were then separated from the glass beads with a pipette and centrifuged.

##### Other Processes

Pestle and mortar can be used to homogenize tentacles for venom studies [9]. Kawabata *et al.* [42] crushed them with PBS. The samples were then centrifuged and the supernatant was filtered through a cellulose acetate membrane filter to obtain the crude extract. A blender can also be used with ice-cold water [46]. In this procedure the crude lysate was clarified through a layer of spun glass and subsequently centrifuged. Using 0.1% trifluoroacetic acid as an incubation solution, Junior *et al.* [45] were able to obtain venom from *Olindias sambaquiensis* tentacles. After freeze-thaw cycles and centrifugation, the supernatant was recovered and filtered through a 0.45 μm filter, followed by a second ultrafiltration using a 0.22 μm filter.

When the whole body of the jellyfish is used to obtain the venom content, mincing can be used to obtaining the venom. Another approach was used by Brinkman *et al.* [16]. For venom collection, Percoll-cleaned nematocysts were washed with Tris-HCl and resuspended in SDS-sample buffer containing DTT (Dithiothreitol) until nematocyst discharge.

The accumulated knowledge revealed the use of glass beads as the most effective approach. Compared to sonication, both techniques require special equipment, but when using glass beads the risk of sample contamination is minor and the time to rupture the cells is less, therefore causing reduced protein degradation when rupturing the nematocysts. The use of a blender or a mortar and pestle also increases the risk of protein degradation and contamination compared to glass beads.

##### Concentration of Venom Proteins

The concentration of total protein in venom extracts can be determined with the Bradford method [19,54], or by measuring absorbance at 280 nm and using bovine serum albumin as a reference standard [15] or by a Pierce BCA assay kit [9,12,13,20,26,46,55]. Alternatively, Nanodrop™ also estimates total protein from extracts [44]. Winter *et al.* [18] have quantified the amount of protein obtained after filtering the supernatant through a micro 0.2 µm syringe filter, read at 562 nm in a Fusion α microplate reader. Other authors quantified the proteins based on the techniques of Waddell [56] or Lowry *et al.* [57]. For the identification and quantification of the different venom components, immunodetection (ELISA and Western blot) or proteomics methodologies combining sample fractionation (liquid chromatography and/or gel electrophoresis) and mass spectrometry could be used. These methodologies are explained in more detail below.

### 2.3. Toxin Purification, Detection and Identification

For toxin purification and identification, several methods can be applied. Sodium dodecyl sulfate polyacrylamide gel electrophoresis (SDS-PAGE), two-dimensional gel electrophoresis (2DE), gel filtration, liquid chromatography-mass spectrometry (LC-MS), reverse-phase high performance liquid chromatography (RP-HPLC), DEAE-Sepharose Fast Flow anion-exchange chromatography, matrix-assisted laser desorption-ionization mass spectrometry (MALDI-MS) are commonly used.

#### 2.3.1. Electrophoresis

Gel electrophoresis allows the discrimination of proteins according to size or purification of a protein extract by picking proteins out of the gel. Briefly, samples are mixed with 4× loading buffer (Tris-HCl, SDS, glycerol, bromophenol blue, β-mercaptoethanol). The samples are reduced at 100 °C and then cooled. The gels of polyacrylamide contained acrylamide/bis solution, Tris-HCl, SDS, TMED and ammonium persulphate. Broad range molecular weight markers are used for quantification. The electrophoretic buffer contains Tris base, glycine and SDS. The protein bands are visualized with Coomassie R-250 staining [15,22], silver nitrate [15,38,39] or both to elucidate any bands that could be missed with just one stain [14]. The gels are then destained in KCl. This SDS-PAGE procedure description follows Laemmli [22,58]. Other authors [23,24] make a SDS-PAGE with slight modifications, namely a sample dilution (*v*/*v* = 1:1) with 2× loading buffer. Prior to 2DE, the venom could be treated with cold acetone for 2 h. The samples are centrifuged and the pellets dried in the lyophilizer. The dried pellets are dissolved in the sample buffer (containing urea, thiourea, CHAPS, DTT and IPG buffer) and afterwards in rehydration buffer (containing the previously described reagents plus bromophenol blue) and then applied to an IPG (Immobilized pH Gradient) strip. After isoelectric focusing (IEF), the IPG strips are first equilibrated with DTT and then with iodoacetamide. Thereafter, the IPG strips are placed onto 12% SDS-PAGE gel and sealed with agarose, before beginning the run [15].

According to several authors, jellyfish venoms assessed by SDS-PAGE [8,9,10,11,12,13,14,15,18,20,24,25,27,33,39,46,47,56] can be analyzed using diverse percentages of polyacrylamide. In a staking gel, it can range from 4% to 5% [10,23,24], and in a separating gel from 10% to 20% [10,15,16,23,24,44]. Gusmani *et al.* [36] made a SDS-PAGE on a continuous gel gradient from 6% to 15% polyacrylamide and Ovchinnikova *et al.* [8] applied a preparative continuous acid-urea-PAGE. Tricine gels are also used to detect smaller-sized proteins compared to glycine gels [14]. A Native PAGE analysis [41,59] can also be made. In this case, the SDS is abolished from the resolving gel and electrophoretic buffer, while the loading buffer lacks β-mercaptoethanol and SDS.

#### 2.3.2. Gel Extraction

Proteins can be obtained through in-gel digestion. Briefly, after electrophoresis each band (SDS-PAGE) or dot (2DE) of interest is excised. In-gel reduction, alkylation, and proteolytic digestion with trypsin are performed for each band/dot gel piece [44]. After digestion, the supernatants are recovered with formic acid and acetonitrile (ACN). The extracts are dried in a vacuum centrifuge [16,25]. Other authors [37,38] digested the venom prior to any separation method. The lyophilized venom is dissolved in guanidine hydrochloride with DTT in Tris-HCl and then reacted at 37 °C. Subsequently, iodoacetic acid is added and reacted at room temperature in the dark. NH_4_HCO_3_ is added and then centrifuged with an ultrafiltration device (Molecular Weight Cut Off—3 kDa). The sample is then digested with trypsin for 20 h at 37 °C. Finally, the digested proteins are lyophilized and stored at −80 °C until use. Brinkman *et al.* [16] used OFFGEL electrophoresis (OGE). Tryptic digested fragments were diluted in peptide-focusing buffer, without the addition of ampholytes. The samples were focused and peptide fractions were harvest and lyophilized.

#### 2.3.3. Gel Filtration and Columns

Gel filtration is a size exclusion technique. It includes media such as Sephacryl and Superose (e.g., 12 HR (high resolution) [36]) for wide ranges of fractionation, Superdex for high resolution, and Sephadex (e.g., G-75 [31]; G-50M [33]) for small molecules in organic solvents.

Sephadex G-50 M equilibrated with acetic acid was used by Sánches-Rodriguez and Lucio-Martinez [33]. Thereafter, the active fraction was passed through a QAE Sephadex A-25 column and subsequently through a Fractogel EMD SO^3−^ column equilibrated with ammonium acetate. Afterwards, the sample was concentrated under vacuum and desalted in a Sephadex G-25 column.

Gel filtration could be a following procedure after ion-exchange chromatography, which allows the separation of proteins based on their affinity to the ion exchanger. Indeed, Feng *et al.* [23] used a Sephadex G-100 column pre-equilibrated with Tris-HCl buffer, Li *et al.* [24] used a Superdex 75 column pre-equilibrated with a size-exclusion standard mixture (SERVA) or a Superdex 200 column [41] and Chaoussis *et al.* [19] used a Superdex 200 10/300 GL Column in Fast Protein Liquid Chromatography (FPLC). This last work [19] wanted to select proteins in a range of 600 kDa–10 kDa, and, therefore, prior to sample running, a standard curve was generated with manufacturer proteins to allow determination of the proteins’ molecular weights. Subsequently, crude venom was dissolved in Dulbecco’s Phosphate Buffered Saline (DPBS) and run through the column for eluting fractions in a 96-well plate. Winter *et al.* [18] used a Superdex S-200 column with PBS solution as the mobile phase. The eluant was monitored at 280 nm. Using similar columns, Radwan *et al.* [47] performed venom elution in a Sephadex G-200 and afterwards in a Sephacryl S-200 HP prepacked column. Proteins were eluted in phosphate buffer. In a different approach, Li *et al.* [39] filtered the solution of interest with a 0.45 µm filter membrane and loaded it onto a Superdex 75 column preequilibrated with NaCl in PBS. The purified protein was subjected to a TSK gel G3000PWxL column. Diaz-Garcia *et al.* [46] purified proteins by resuspending the lyophilized crude extract in ammonium acetate following filtration through a 0.22 µm membrane. Aliquots were subjected to gel filtration on a Superdex 75 column. The column was previously equilibrated and eluted with the sample buffer. The low molecular weight fractions of interest were pooled and loaded onto a Phenomenex ODS-3 reverse phase column. Components were eluted using a linear gradient of ACN:TFA (acetonitrile:trifluoroacetic acid). All columns mentioned were connected to a HPLC system.

#### 2.3.4. Fast Flow Anion-Exchange Chromatography

DEAE-Sepharose Fast Flow anion-exchange chromatography is a technique frequently employed for toxin purification. Feng *et al.* [23] pre-equilibrated the column with Tris-HCl buffer, and the nematocyst extract was ultrafiltered using a 10 kDa membrane. The column was stepwise-eluted using a NaCl gradient ranging from 0.1 to 0.6 M in the equilibrating buffer. Fractions showing lethal activities in the bioassay were collected, pooled, concentrated by ultrafiltration and proceeded to gel filtration. For isolation of the hemolytic proteins from the nematocyst venom of the jellyfish *Stomolophus meleagris* [41], the hemolytic fractions of the dialyzed venom sample were loaded onto a 60 mL DEAE Sepharose Fast Flow column. Afterwards, the eluates were pooled and concentrated by a 3 kDa ultrafiltration device and went for a gel filtration. In another study, Li *et al.* [24] applied the crude venom to a 16/20 ion-exchange chromatography column packed with DEAE-Sepharose Fast Flow, which was pre-equilibrated and connected to a UV-ribonucleoprotein detector. After removing unbound proteins with EBS, the column was then eluted using stepwise increases in NaCl in Tris-HCl. The eluted fractions were monitored by UV detection and were then collected. Fractions of the same peak were pooled and concentrated using a 3 kDa Ultra centrifugal filter device and subjected to a hemolytic activity test. Thereafter, the toxic fractions were purificated by gel filtration.

#### 2.3.5. HPLC

HPLC is a technique that purifies more or less complex protein samples. It can be used as one of the first techniques for purifying proteins or it can be used after more coarse methods, such as gel filtration and toxicity assays, as explained in the following examples. Ponce *et al.* [9] performed a chromatography fractionation of the venom in a Vydac C18 analytical RP-HPLC column. Maisano *et al.* [27] inserted its extracts in a BioSuite 250, 10 µm SEC, 7.5 × 300 mm column. Nagai *et al.* [12,13,20,55] applied venom extracts first to an ion-exchange HPLC, TSK-GEL CM-650S column and then to a TSK-GEL CM-5PW column. Throughout the purification process, each fraction was checked for its hemolytic activity and pooled. The concentrated sample was applied to a gel-permeation HPLC, Superdex 75 column. Sánchez-Rodríguez [33] determined the purity of the protein fraction by HPLC on a Varian ProStar 410 Autosampler using a Nucleosil C18 reversed phase column. Ovchinnikova *et al.* [8] purified by RP-HPLC on a Macrosphere 300 C-18 column, with active fractions in microbes. Nagai *et al.* [13] incubated the toxins first with lysylendopeptidase and then applied the samples onto a reversed-phase HPLC column: Bakerbond wide-pore ODS 5 µm with MeCN:TFA as solvent system.

#### 2.3.6. Mass Spectrometry

Besides HPLC, nano liquid chromatography (nanoLC-MS/MS) has been also successfully applied in other studies. LC-MS/MS is one of the most powerful techniques in the field of proteomics, allowing high-throughput identification of proteins out of complex protein mixtures. Li *et al.* [37,38], for the identification of venom extracted from the jellyfish *Stomolophus meleagris*, desalted the digested proteins on reversed phase columns (Zorbax 300 SB C18), which were then separated with an analytical RP (Reverse phase) column using the Ettan MDLC system. The process of separation used a Finnigan LTQ linear ion trap MS equipped with an electrospray interface connected to the LC setup for eluted peptide detection. Brinkman *et al.* [16], for protein identification, used a Dionex Ultimate 3000 HPLC with an Agilent Zorbax 300SB-C18 column. Eluates from the RP-HPLC column were directly introduced into the NanoSpray II ionization source of a QSTAR Elite Hybrid MS/MS System operated in positive ion electrospray mode. Weston *et al.* [44] reconstituted the protein extract in ammonium bicarbonate prior to LC-MS/MS analysis and samples were analyzed on a Thermo Scientific Orbitrap Velos Pro mass spectrometer coupled to an EASY-nLC II (Proxeon) nano LC system. After mass spectrometric analysis, liquid chromatographic separation was performed. Samples were trapped on an Easy-column packed with ReproSil-Pur C18 (3 µm). In these procedures the separations were performed with a gradient of formic acid: ACN.

MALDI-ToF allows the identification of peptides and is commonly applied to jellyfish venom analysis [25]. Li *et al.* [39] made a Peptide Mass Fingerprinting (PMF) analysis using a MALDI-ToF-MS, where the *N*-terminal amino acid sequence was determined by the method of Edman degradation. Diaz-Garcia *et al.* [46] used this same method and an automatic gas-phase protein sequencer. Another example of the application of this technique is the peptide molecular mass determination described by Ovchinnikova *et al.* [8], where peptide microsequencing was made in the Procise cLC 491 Protein Sequencing System. Likewise, Brinkman *et al.* [15] determined the internal amino acid sequences for two jellyfish toxins. Nagai *et al.* [13] used this technique for peptide mapping of toxins, where separated peptide fragments were fractionated and their amino acid sequences analyzed by a PSQ-1 protein sequencer.

For protein purification, detection and identification, gel electrophoresis is a technique transverse to all types of works. It does not require expensive equipment, and is fast and most informative. For the separation of toxin fragments, gel filtration is one of the most employed and easy-to-use techniques. Other methodologies referred to here require more specialized skills and their selection should be based on study purpose and easy access to that equipment.

#### 2.3.7. Glycoproteins, Phosphoproteins and Antioxidant Protein Detection

Brinkman *et al.* [16] were able to detect glycoproteins and phosphoproteins using a GlycoProfile III fluorescent glycoprotein detection kit and a Pro-Q Diamond phosphoprotein gel stain, respectively. For glycoprotein analysis, a duplicate gel was processed, omitting the oxidation step to detect any non-specific fluorescent staining. For phosphoprotein analysis, the ProteoProfile PTM marker containing phosphorylated ovalbumin and β-casein was included as a positive control. Fluorescently stained glycoproteins and phosphoproteins were visualized using a ChemiSmart 3000 image acquisition system.

For the purification of an antioxidant protein, Li *et al.* [39] used an ammonium sulfate precipitation procedure. For that purpose, the nematocysts were sonicated in cold extraction buffer with sodium EDTA, PMSF, pepstatin A, leupeptin and aprotinin in PBS. Ammonium sulfate was added into aliquots of seven groups representing saturation from 20% to 80%. The precipitation was dissolved and dialyzed for further analysis [39].

#### 2.3.8. Western Blot Analysis

In a *Chironex fleckeri* study [15] to detect the proteins of interest (CfTX-1 and CfTX-2), as well as in other works [16,25], a Western blot analysis was performed using polyclonal antibodies purchased or raised in rabbits. The nematocyst extract proteins separated by SDS-PAGE were transferred to Immobilon-P^®^ membranes. Membranes were blocked in non-fat milk powder in TBST and incubated overnight with either purchased antibodies or rabbit antiserum diluted in blocking solution. Membranes were washed in TBST, then incubated with secondary antibodies, conjugated to alkaline phosphatase and diluted in TBST. Following membrane washing, antigenic proteins were visualized using NBT/BCIP [15]. The antibody-containing serum was analyzed by ELISA assay [25]. Nagai *et al.* [13] also completed their study with a Western immunoblotting assay. The blots were saturated with a 5% skim milk solution in phosphate buffer containing Tween 20, and reacted with the polyclonal antiserum against the toxin-fragmented peptide. The bands were revealed using a peroxidase-conjugated anti-rabbit serum and the Western blotting detection reagent ECL Plus.

### 2.4. Toxicity Assays and Others

#### 2.4.1. Venom Proteolytic Activity

Gusmani *et al.* [36] and Lee *et al.* [11] assessed the proteolytic activity of jellyfish venom with gelatin, casein, and fibrin as substrates that were dissolved in sodium phosphate buffer and used in 15% polyacrylamide zymography gels. Venom extracts were prepared in non-reducing sample buffer, and then run on gels at 4 °C.

After electrophoresis, SDS was removed by washing the gel twice in Triton X-100. The gel was incubated in Tris and calcium chloride and stained with Coomassie blue. Clear zones in the gel indicate regions of proteolytic activity. When required, the protease inhibitor 1,10-phenanthroline was added to the wash and incubation buffers, and to the stained gel. For hyaluronidase activity, the hyaluronic acid was used as a substrate in SDS-PAGE following Miura *et al.* [60]. Residual proteins (which may interfere with gel staining) were removed by adding *S. griseus* protease and incubating. The gel was stained with Alcian blue and the caseinolytic activity was also measured. An aliquot of casein (1%, in potassium phosphate buffer) substrate was incubated for 3 h in the absence (as blank) or the presence of jellyfish venom. The reaction was stopped by the addition of trichloroacetic acid and the casein hydrolysis was measured by modifying the Folin-Ciocalteu method. For the blank, the same amount of venom was added before the measurement. A standard graph was generated using standard tyrosine solution. The developed colors of reaction mixtures and standard mixtures were read at 660 nm. For the inhibition study of caseinolytic activity, metalloproteinase inhibitors (EDTA, EGTA, 1,10-penanthroline) and a serine proteinase inhibitor (PMSF) were used.

The venoms were preincubated with an inhibitor, and then its caseinolytic activity was measured as previously described. One unit of the caseinolytic activity was defined as the amount of enzyme, which hydrolyzed casein to liberate 1 mg of tyrosine per minute. Li *et al.* [40] studied the protease activity of venom from *Rhopilema esculentum* using the Folin-phenol of the Bakhtiar method [34]. Briefly, casein in sodium phosphate buffer (PBS) and crude protein were preincubated. Then unhydrolyzed protein was precipitated with trichloroacetic acid and softly shaken. After centrifugation, Na_2_CO_3_ was added to the supernatant followed by the Folin-phenol reagent and then immediately shaken up. Absorbance at 640 nm was measured for the reaction mixture. One unit of protease activity was defined as 1 ng tyrosine released from casein hydrolyzed by protease of 1 mL of crude protein at 37 °C, pH 8.0, for 1 min. The effects of temperature, pH and additives on protease activity were also assessed.

#### 2.4.2. Hemolytic Assay

The hemolytic assay is commonly used to determine if the protein solution has toxic properties such as the hemolysis of cells [12,13,20,24,36,40,41,55]. Yu *et al.* [35] studied the hemolytic activity of the *Rhopilema esculentum* venom according to the method previously described for *C. marsupialis* [61]. Briefly, 0.5 ml of a 0.05% suspension of erythrocytes in Krebs Ringer phosphate buffer (KRP), pH 7.4, was incubated at 37 °C for 30 min with different amounts of venom. After centrifugation, the hemolytic activity was evaluated spectrophotometrically at 415 nm by assaying the hemoglobin released in the supernatant. Reference samples were employed using hypotonic lysis with water as a 100% lysis reference and the supernatant of 0.05% erythrocyte suspension (0.5 mL) incubated with 4.5 mL KRP at 37 °C for 30 min as the 0% reference. The HU_50_ was defined as the amount of venom required to cause 50% lysis. Junior *et al.* [45] also made a hemolytic assay slightly modified from the previous: human blood, freshly collected with heparin, was centrifuged to remove the buffy coat, and the erythrocytes obtained were washed three times in 0.85% saline and stored at 4 °C. Toxins at desired concentrations were added in the first well to erythrocyte buffer, and then were serially diluted in a two-fold ratio. Red blood cells in erythrocyte buffer were added to the toxins, and hemolysis was monitored by measuring attenuance at 630 nm for 20 min at room temperature. The final volume was 200 μL per well. The hemolysis percentage was determined at the end of the assay.

#### 2.4.3. Antimicrobial Assay

Ovchinnikova *et al.* [8] developed an antimicrobial assay to assess venom toxicity activity. Antimicrobial activities of peptides were measured in radial diffusion assays by the agarose gel overlay technique according to Lehrer *et al.* [62]. Samples were tested against *E. coli* and *Listeria monocytogenes*. The microorganisms were precultured in tryptic soy broth at 37 °C for 16 h. Aliquots of the bacteria-containing medium were transferred into a freshly prepared medium and incubated at 37 °C for 2.5 h to obtain mid-logarithmic phase microorganisms. Cell counts for each microorganism were determined, using a spectrophotometer, by the turbidity of cell suspensions at 620 nm. Suspension aliquots were mixed with sterile agarose solution in sodium phosphate buffer and poured into sterile plastic 90 mm Petri dishes. Wells made by a 3 mm applicator were filled with 5 µL test samples. The diameter of the inhibited growth zone (the microbe-free zone around a well) was measured assuming 0.1 mm as one unit of antimicrobial activity and subtracting 30 such units (the well diameter) from each result.

## 3. Genomics/Transcriptomics

The genetic code of organisms can be used for unraveling a variety of biological processes. Deep sequencing technologies generate genomic and transcriptomic sequences [63]. Several fields benefit from deep sequencing such as evolutionary [64], bioprospecting [37,65] or metagenomic (environmental genomics describing highly diverse microbial communities) studies [66]. In brief, next generation sequencing (NGS) technology has facilitated genome re-sequencing, *de novo* genome assembly, transcriptome and non-coding RNA sequencing, transcriptome assembly, as well as sequencing of genome-wide protein-binding or methylation sites (ChIP-seq and Methyl-seq) [67]. Sequencing can include a whole individual [65], a pool of individuals [68], a specific tissue [37] or even a microbial community associated with an organism, such as the microbial communities investigated by 454 pyrosequencing in cnidarians and sponges [69,70]. Deep sequencing or NGS or high-throughput sequencing are all synonyms. Deep sequencing for transcriptome analysis, also referred to as RNAseq [71], will be considered in further detail in this review. The transcriptome represents the full complement of RNA transcripts expressed in a cell for a specific developmental stage or physiological condition, and consists of protein-coding RNA transcripts (mRNAs) and non-protein-coding RNA transcripts. Only a fraction of the total cellular RNA is referred to as mRNA, which is often in very low abundance and requires extremely sensitive analytical tools to be identified and characterized. RNAseq allows scientists to look at different populations of RNA such as total RNA, miRNA, tRNA [72], alternative gene spliced transcripts, post-transcriptional modifications, gene fusion, mutations/SNPs (Single Nucleotide Polymorphisms) [73] and it even allows them to determine exon/intron boundaries and verify or amend previously annotated 5ʹ and 3ʹ gene boundaries. Overall, the key aims of transcriptomics are to obtain an exhaustive catalogue of transcripts, allowing the determination of the transcriptional structure of genes and the quantification of changes in expression levels among transcriptome samples [3].

### 3.1. Wet-Lab Genomics for Toxin-Coding Gene Discovery

After the separation of the toxic fraction from the crude extract, it is a common aim to characterize the amino acid sequence of the relevant proteins and the DNA sequencing of related protein-coding genes. Relative to the number of proteins identified in sea anemones’ venoms, and therefore those sequenced [6], jellyfish have considerably reduced figures even though their proteins are supposedly more toxic. Table 1 summarizes the toxins identified in jellyfish and the available amino acid (aa) and nucleotide (nt) sequences, or if information only exists on protein mass or bioactivity (-).

The work of Lassen *et al.* [74] is an example of the effort placed to obtain the amino acid sequence of CcTX-I, a toxin from *Cyanea capillata*, but not the protein-coding gene sequence. This was determined by MALDI-ToF/ToF MS/MS *de novo* sequencing after a size-exclusion, cation-exchange and reversed-phase chromatography.

Nagai *et al.* [12,13,20] provided one of the first works that discovered the gene encoding for jellyfish toxins. First they isolated the toxin by HPLC, complemented with SDS-PAGE and Western blotting. Then they cloned it and performed a Northern analysis. For that purpose, total RNA was isolated from the intact tentacles using TRIzol^®^ in a bead mill or a mortar and pestle. First-strand cDNA was synthesized from the total RNA using SuperScript^®^ II reverse transcriptase and an oligo (dT) 12–18 primer. A degenerative reverse transcriptase-polymerase chain reaction (RT-PCR) using mixed oligonucleotide primers was performed to obtain a partial cDNA fragment. Mixed oligonucleotide primers were designed based on the amino acid sequences of the peptides. Amplification was carried out using Ex *Taq* polymerase and all possible combinations of the primers were used. The obtained products were cloned into a vector and nucleotide sequences were determined using the BigDye^®^ Terminator Cycle Sequencing Kit. The cDNA was then subjected to a 5′/3′–rapid amplification of cDNA ends (RACE), using a 5′/3′–RACE kit. The secondary PCR products were subcloned into a vector and sequenced. Full-length cDNA was recloned directly from the total RNA by RT-PCR. PCR products were subcloned and nucleotide sequencing was carried out as described above. Northern analysis was processed as follows: total RNA was separated on a denaturing 1% agarose-formaldehyde gel. Separated RNAs were transferred and crosslinked onto a nylon membrane GeneScreen Plus^®^. The full-length cDNA of the toxin was labeled with DIG DNA Labeling Kit as a probe. Hybridization and detection were carried out according to the DIG system. The antibodies were raised in rabbits.

Similarly to the previous works, Ovchinnikova *et al.* [8] determined the nucleotide sequence of aurelin, a protein from *Aurelia aurita*. They used a SV Total RNA Isolation System and a SMART™ RACE cDNA Amplification Kit. Likewise, Brinkman and Burnell [15] screened the gene encoding for a *Chironex fleckeri* toxin, using a bead mill, TRIzol^®^, a MicroPoly (A) Pure™ Kit, a ZAP-cDNA Gigapack II Gold Cloning Kit and Ready-to-Go RT-PCR Beads.

### 3.2. Deep Sequencing Platforms

Deep sequencing is an outstanding improvement in the discovery of new bioactive molecules, apart from all the other advantages previously mentioned. The sequencing of these millions of databases generates thousands of data that are stored in large electronic archives and processed into gene product profiles (mainly proteins, peptides and RNAs) [1,3]. The *in silico* analysis is another approach in bioprospecting, contrasting with the proteomic version that we explained previously. By sequencing genes, genomes and transcriptomes, the search for gene homologs, motifs or transcripts with a certain expression profile can be undertaken [1]. Deep sequencing has one major advantage (beside others), as its relative low cost allows the sequencing of whole transcriptomes of non-model organisms in a relatively short time period [1]. Fischer *et al.* [98] constructed a database only for transcriptomes of marine organisms with the purpose of sharing and searching transcriptomic data, a major tool for identifying mechanisms of development and evolution, regeneration, resistance to cancer, longevity and symbiosis, among others. This database can be accessed online at: http://seabase.core.cli.mbl.edu/. However, currently only the transcriptome of *Nematostella vectensis* is available [98]. Further information on this species genome can be retrieved from the works of Moran and co-workers [99,100,101].

Several deep sequencing technologies are available such as Capillary Sequencing ABI, Ion Torrent, 454 Pyrosequencing from Roche, the Genome Analyzer platform from illumina Sequencing technologies and the SOLiD (Sequencing by Oligonucleotide Ligation and Detection) from Life Technologies, among others. All of these are multi-step processes that differ in sample preparation, sequencing methods [3,66,71,102,103,104,105,106,107,108], mapping tools [105] and also by the type of errors they generate [63,66]. The last three deep sequencing platforms have dominated whole transcriptome analysis [1].

In practical terms, SOLiD sequencing is highly preferred in small RNA and gene expression profiling, as well as in whole transcriptome re-sequencing [103]. For example, it is suitable when aiming to identify a particular mRNA that translates into a toxin. In contrast, 454 gives longer reads (700 bp) [105,108]. In the absence of an annotated sequenced genome, Siebert *et al.* [105] showed that a hybrid long-read/short-read sequencing strategy is an effective way to collect gene expression data. As mentioned above, this is the case for the large majority of jellyfish species. Moreover, assembling raw sequence reads into a reference of gene sequences is best served by long reads, but quantifying gene abundance is easily accomplished by having many reads. However, it is less expensive to collect short reads than long reads. Thus, collecting long reads across all the samples to be analyzed (including multiple treatments and biological replicates) would therefore greatly increase the project cost or greatly reduce the number of reads that could be sequenced for quantification [105,108]. Illumina and short-read sequencing, in general, may be a more appropriate method for metagenomic studies. Roche 454 may be advantageous for resolving sequences with repetitive structures or palindromes or for metagenomic analyses based on unassembled reads, given the substantially longer read length. Nonetheless, both platforms (454 and illumina) provide comparable results [66].

After obtaining the digital data, there are two main different assembly methods for producing a transcriptome from raw sequence reads: *de novo* and genome-guided. *De novo* can be done with special software such as Trans-ABySS, SOAPdenovo, Velvet/Oases, or Trinity [109]. The other approach, “easier” and relatively computationally cheaper, is to align the millions of reads to a “reference genome” using, for example, Geneious or CodonCode Aligner software. This, however, has a major problem because it depends on the quality of the reference genome [109]. Moreover, there is the problem of reads that align equally well with multiple regions of the genome. The program must then choose if these reads are excluded, which can result in gaps, or it will decide which alignments should be retained, which could lead to wrong assignments or incorrect predictions of transcripts [109].

**Figure 1:**
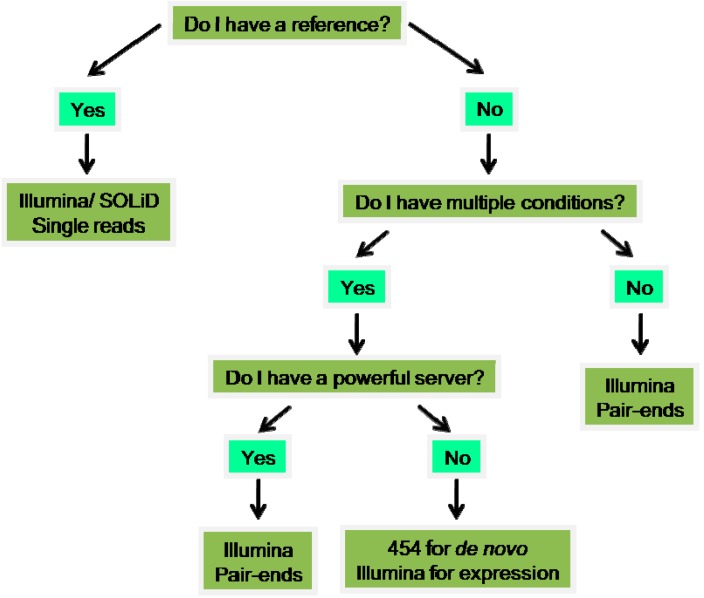
Selecting the right sequencing technology (adapted from [108]).

**Figure 2:**
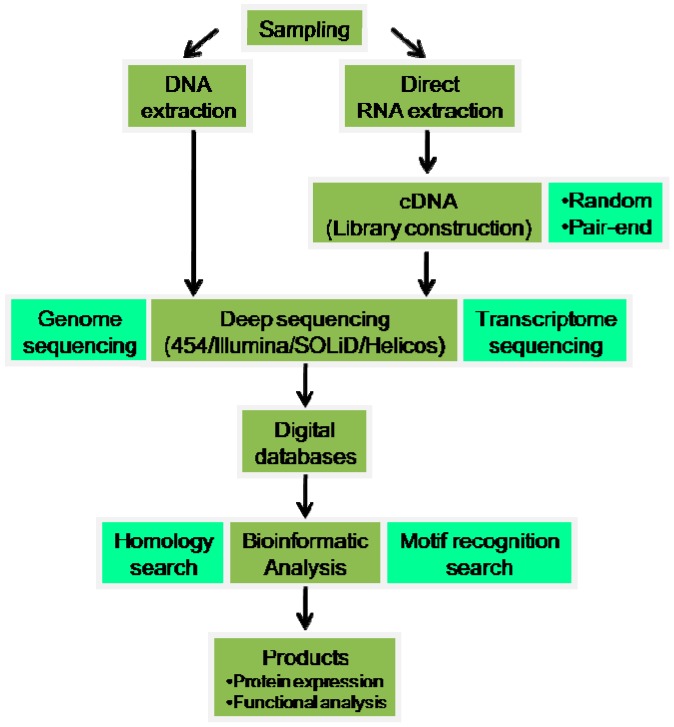
Deep sequencing workflow (adapted from [1]).

Another feature that researchers have to choose, beyond the platform choice, is the kind of reads that they want to analyze: pair-ends or single-ends. Paired-end (PE) sequencing allows users to sequence both ends of a fragment and generate high quality, alignable sequence data. This facilitates the detection of genomic rearrangements and repetitive sequence elements, as well as gene fusions and novel transcripts. Single-ends (SE) involves sequencing DNA from only one end. This solution is more rapid and affordable. Moreover, it has the advantage of avoiding homopolymer sequencing errors and the G-C bias [109,110]. Figure 1 schematically condenses the information on deep sequencing technologies, facilitating the selection of the best approach as a subject of study. Figure 2 represents a diagram to visualize the deep sequencing workflow.

As an emergent technology, deep sequencing is not supported by the same volume of published work as compared to Sanger sequencing technology. Among cnidarians, there are already several works on sea anemones, corals, Hydrozoa and just one in jellyfish, including the transcriptome of *Stomolophus meleagris* (obtained with illumina HiSeq™ 2000) (see Table 2).

Concerning jellyfish genome deep sequencing, there are more works, but all refer to mitochondrial genomes. *Alatina moseri* (*Carybdea alata)* was sequenced by Roche 454 (GS FLX Titanium) and ABI SOLiD [111]. *Lophelia pertusa* was sequenced by by SOLiD [112] and *Cassiopea andromeda*, *Carybdea xaymacana*, *Cassiopea frondosa*, *Chrysaora* sp. *Carukia barnesi*, *Chironex fleckeri*, *Alatina moseri*, *Chiropsalmus quadrumanus*, *Cyanea capillata*, *Nemopsis bachei*, *Catostylus mosaicus*, *Linuche unguiculata*, *Rhizostoma pulmoa* and *Pelagia noctiluca*, with nearly complete mitochondrial genomes sequenced [113], were done by the Sanger method and long PCR using 454 high throughput or illumina Sequencing platforms.

### 3.3. RNA Procedures

Before performing deep sequencing, wet-lab procedures must be undertaken for extracting and preparing RNA. In this review we do not approach the techniques employed for Sanger sequencing. Beginners should have a good background in working with DNA before working with RNA, which is very sensitive to heat degradation and contamination. Therefore, having an isolated physical space in the lab is advisable for RNA manipulation. Moreover, special materials, such as gloves, tips and reagents, among many others, should be RNAse free and should be considered for RNA-restricted use.

The RNA extraction is performed with two main objectives: for Rapid Amplification of cDNA Ends (RACE technology), which aims for the discovery of a toxin-coding gene sequence, or for large-scale sequencing to unravel new bioactive compounds. This review describes both technologies. The first was already described in Section 3.1.

As an example of large-scale sequencing, Li *et al.* [37] sequenced the venom gland of the jellyfish *Stomolophus meleagris* on illumina HiSeq™ 2000. Several procedures were conducted on short mRNA fragments that were used as templates. Random hexamer-primer was used to synthesize the first strand of the cDNA. The second strand of the cDNA was synthesized using buffer, dNTPs, RNaseH and DNA polymerase I. The short fragments were purified with a QIAQuick^®^ PCR extraction kit and resolved with EB buffer to end reparation and a polyA tail was added. The suitable fragments with sequencing adaptors were selected as templates for PCR amplification based on the agarose gel electrophoresis results. The samples were clustered in flow cells to construct the cDNA library and loaded onto the platform for sequencing.

As only one jellyfish transcriptome is currently available [37], we refer to similar works on sea anemones, which would allow us to establish a parallel. *Bunodosoma granulifera* [65] and *Aiptasia* sp. [115] were sequenced by 454 and illumina, respectively. RNA was extracted using TRIzol^®^ and purified using RNeasy Mini Spin Column. A high-salt method of RNA precipitation can also be used to reduce proteoglycan and polysaccharide contamination. DNA digestion is performed using a DNase. The quality and quantity of the total RNA was detected using the RNA 6000 pico LabChip^®^ kit. The cDNA library was prepared with kits compatible with the platform in use. *Nematostella vectensis*, a model system for studying the evolution of animal body plans, has had several works preformed, all with illumina HiSeq [68,109,116]. Total RNA was extracted with a Tri-reagent kit and purified with the RNA Clean & Concentrator™ kit, or with the mRNA DIRECT™ kit. Dynabeads and low-adhesion microcentrifuge tubes were used. Genomic DNA residues were removed by DNase. RNA concentrations were further determined in a NanoDrop™ or in a Qubit^®^. Samples were prepared for multiplex sequencing using illumina TruSeq kits [68,109,116].

The various deep sequencing technologies share the massive sequencing of DNA in a flow cell. The flow cell is a glass slide with one, two, or eight physically separated lanes, where the sample is loaded. Helm *et al.* [68] sequenced in a single lane. Elran *et al.* [116] sequenced triplicates of the samples in two lanes. In the work of Tulin *et al.* [109], the RNAseq Library was prepared with the ScriptSeq™ v2 kit using Phusion High Fidelity polymerase with bar-coded illumina-compatible primers. The libraries were size selected for 450 bp. The samples were run on a single lane.

## 4. Conclusions

Bioactive components from jellyfish can be characterized by various techniques. The traditional approach uses the venom extract followed by purification procedures until obtaining more or less pure toxins. Examples of such trials include the SDS-PAGE, 2DE, gel filtration, LC-MS, RP-HPLC, DEAE-Sepharose Fast Flow anion-exchange- chromatography, MALDI-ToF MS, *etc*. Prior to purification procedures, most techniques for venom extraction employ nematocysts that are removed from the tentacles by overnight agitation in seawater. Nonetheless, freshwater can also be used. The rupture of nematocysts can be accomplished with a mortar and pestle, bead mills grinding in an electrical pulverizer, or even point tip sonication. Beside these techniques, venom can be obtained by a chemical discharge of the nematocysts using buffers such as glycerin or sodium citrate. All the procedures should be made on ice and the samples should be stored preferentially at −80 °C, as toxins are heat-sensitive. Protein quantification can be made by various methods, but the Bradford method is well established among scientists. After isolating the toxic fraction/protein, several assays can be considered to decipher the toxin modes of action. There are proteolytic, hyaluronidase and caseinolytic activity assays, as well as hemolytic and antimicrobial assays.

Amino acid sequencing determination is made in MALDI-ToF by the Edman degradation method. The finding and study of the mRNA translating the toxin is performed after the identification and characterization of the toxins by Western blotting, RACE, RT-PCR, cloning and Northern analysis.

Currently, deep sequencing of a species transcriptome is emerging as a valuable approach for bioprospecting molecules *in silico*. For jellyfish, only one work previously used the illumina platform. However, other platforms such as 454, illumina, SOLiD and Helicos have been successfully used in other cnidarians.

Even with so many techniques available, major challenges still remain, such as the complexity of the sample, the scarcity of the biological material, and the absence of databases for the determination of peptide and protein sequences in jellyfish [127].

## Figures and Tables

**Table 1:** Jellyfish toxins described to date. Toxins that have amino acid (aa) or nucleotide (nt) sequences described are labeled in the table. Toxins with only molecular mass or bioactivity action described are labeled with a dash.

Image	Species	Toxin	Sequence	Reference
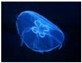 [75]	*Aurelia aurita*	Aurelin	nt	[8]
		Metalloprotease	-	[11]
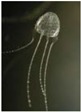 [76] *	*Carukia barnesi*	CbTX-I	nt	[77]
		CbTX-II	nt	
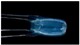 [78]	*Carybdea alata*	CaTX-A	nt	[12]
		CaTX-B	nt	
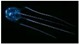 [79]	*Carybdea rastoni*	CrTX-A	nt	[13]
		CrTX-B	nt	
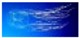 [80]	*Chironex fleckeri*	CfTX-1 CfTX-2 CfTX-A CfTX-B	nt nt nt nt	[15,81] [82]
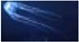 [83]	*Chiropsalmus quadrigatus*	CqTX-A	nt	[20]
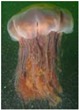 [84]	*Cyanea capillata (Cyanea nozakii)*	CcTX-1 CcNT Metalloprotease	aa -	[74] [85] [11]
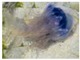 [86] *	*Cyanea lamarckii*	ClGP-1	-	[87]
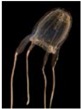 [88] *	*Malo kingi*	MkTX-A MkTX-B	nt nt	[77]
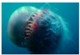 [89]	*Nemopilema nomurai*	Metalloprotease	-	[11] [25]
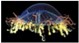 [90]	*Olindias sambaquiensis*	Metalloprotease	-	[44]
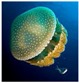 [91]	*Phyllorhiza punctata*	Saxitoxin Gonyautoxin-4 Tetrodotoxin Brevetoxin-2	- - - - -	[92]
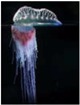 [93]	*Physalia physalis*	Physalitoxin P1 P3 PpV9.4	- - - -	[94] [95] [96] [46]
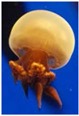 [83]	*Rhopilema esculenta*	Metalloprotease	-	[11]
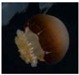 [97]	*Stomolophus meleagris*	SmP90 C-type lectin, PLA_2_, K_v_^+^ toxin, Hemolysin Metalloprotease	aa - - -	[39] [37]

* Permission was granted by the original authors.

**Table 2:** Deep sequencing platforms used in Cnidaria. For each species, the table identifies its order, the tissue used, the number of raw reads obtained and its length and reference. In the Order column, pictures labeled with an asterisk (*) identify a given species mentioned in the corresponding Species column. The information on paired-end (PE) or single-end (SE) is placed in the raw reads column when mentioned in the reference paper.

Order	Species	Tissue	Sequencing Platfform	Raw Reads (Milions)	Read Lengh (bp)	Reference
Actiniaria 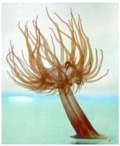 * [114]	Aiptasia palliada *	Adults growing under different conditions	illumina	208	-	[115]
	*Bunodosoma granulifera*	Adult	454	-	-	[65]
	*Edwardsiella lineata*	five developmental stages	illumina	376.2 PE	40	[64]
	*Nematostella vectensis*	six developmental stages Adult stress w/four heavy metals	illumina	165 SE 200 PE 15.22 SE	50 100	[68] [109] [116]
Scleractinia 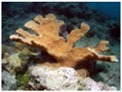 * [117] ^a^	*Acropora palmata* *	Larvae	454 GS-FLX	0.960	398	[118]
	*Acropora millipora*	Larvae w/CO_2_ stress Larvae	illumina 454 GS-FLX	28 628 PE	38 232	[119] [120]
	*Favia corals*	Adult	illumina	80 PE	75	[121]
	*Stylophora pistillata*	Adults growing under different conditions	454 GS-FLX	521	-	[122]
	*Pocillopora damicornis*	Adult colonies subject to a battery of stressors	454	0.955	379	[123]
	*Platygyra carnosus*	Adult colonies	illumina	83 PE	90	[124]
Hydrozoa 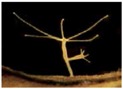 * [125] ^a^	Hydractinia symbiolongicarpus	Adult feeding, reproductive, and defensive polyps	illumina	0.066	200	[126]
	Hydra vulgaris *	Regenerating polyps	illumina 454 Titanium	53.6 1.2	-	[63]
	*Nanomia bijuca*	Nectophores, gastrozooids	454, illumina, SOLiD SAGE, Helicos DGE	943	-	[105]
Scyphozoa 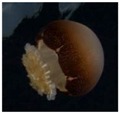 [97]	*Stomolophus meleagris*	Tentacles	illumina	108	90	[37]

^a^ Permission was granted by the original authors.
